# Supplementary material for: Development of Clinical Radiomics-Based Models to Predict Survival Outcome in Pancreatic Ductal Adenocarcinoma: A Multicenter Retrospective Study
Source: Diagnostics (Basel). 2024 Mar 28;14(7):712. doi: 10.3390/diagnostics14070712 (PMC11011556; doi:10.3390/diagnostics14070712)
Supplement: Supplementary file 1 [file diagnostics-14-00712-s001.zip › Diagnostics - Supplementary Materials-for proof.pdf]

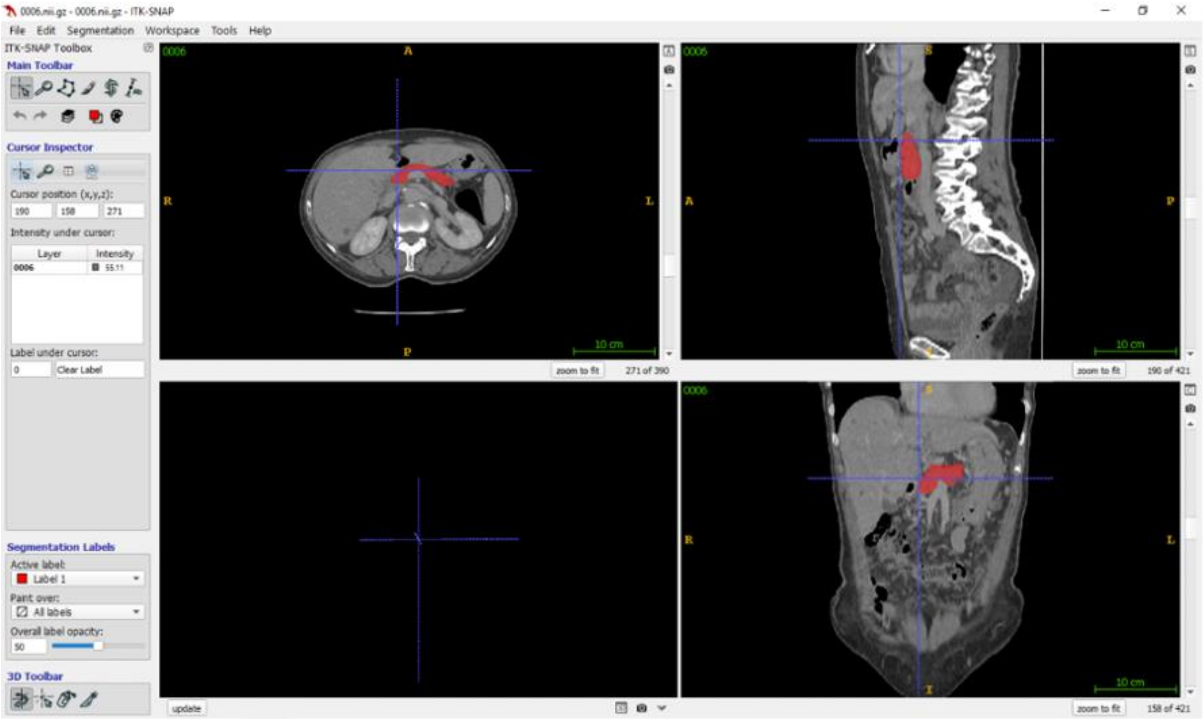

Figure S1: Example of automated segmentation for RPV using ITK-SNAP.

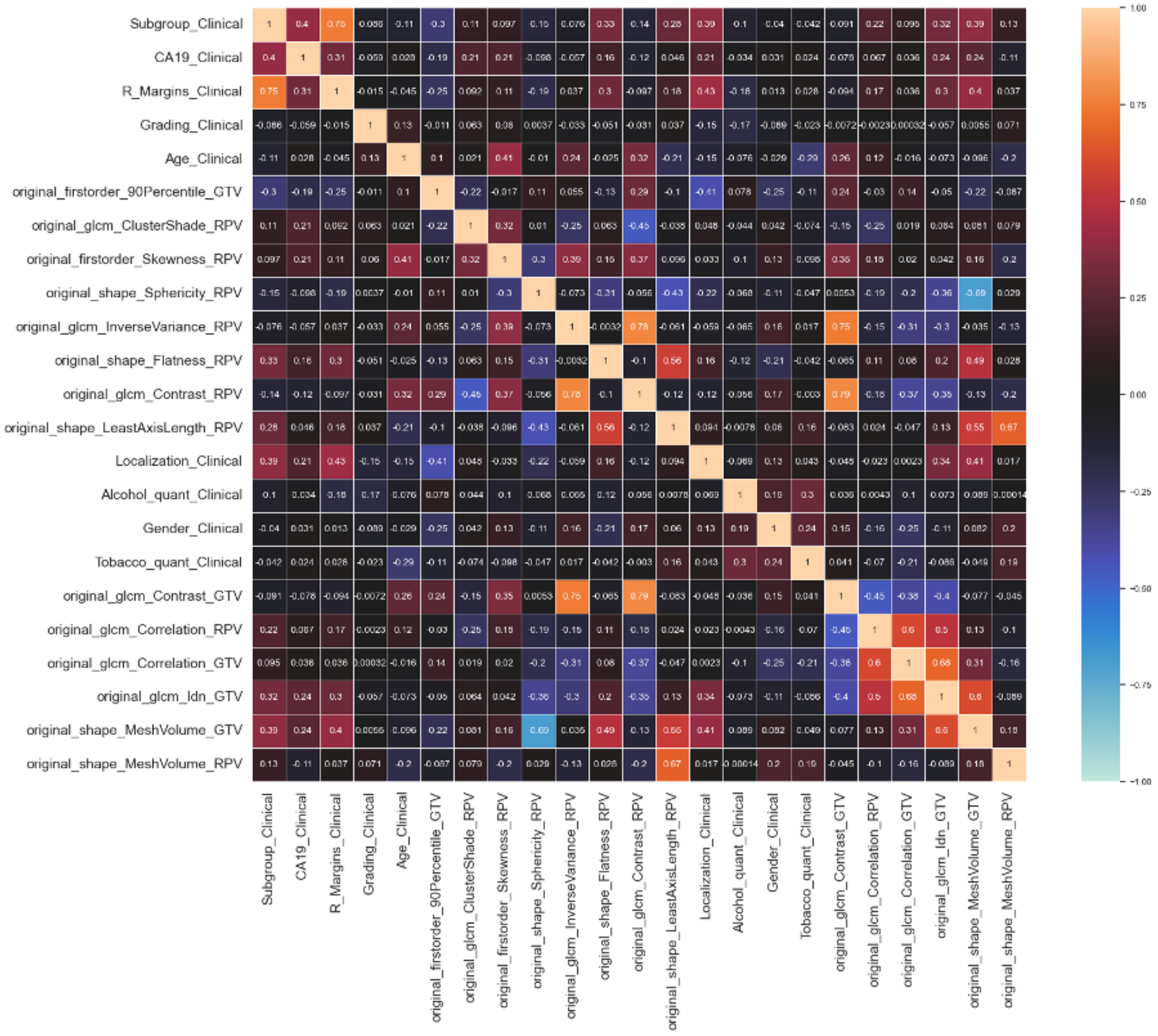

Figure S2: The intercorrelation between the chosen features in the testing dataset (missing clinical features were filled using the MissForest package; the values were not standardised).

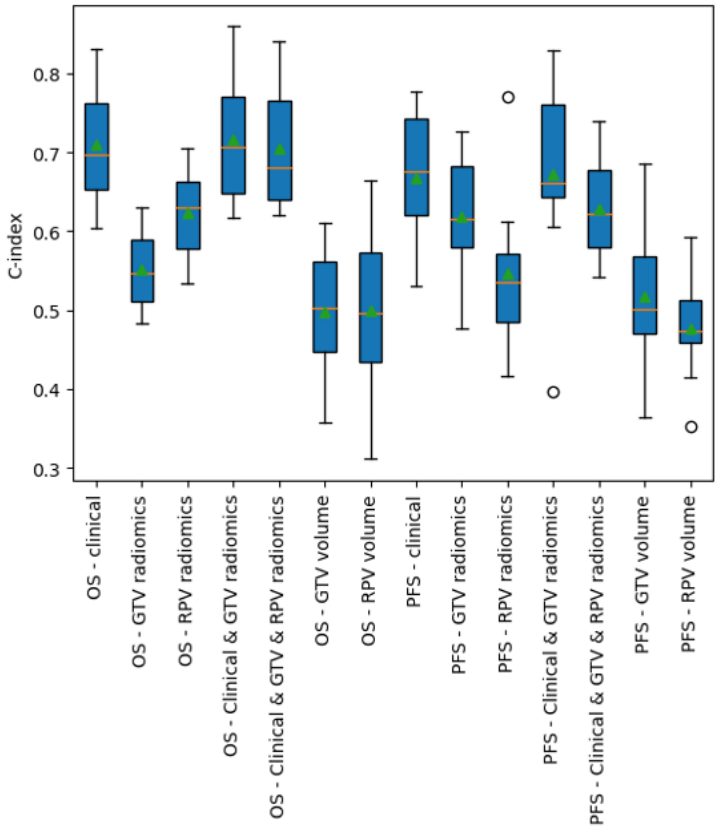

Figure S3: Boxplot showing the comparison of different models (training-validation dataset 10-fold Cross Validation). The blue central boxes represent the interquartile range, with the median indicated by horizontal orange lines inside. Whiskers extend to the maximum non-outlier data points, circles denote outliers, and green triangles indicate the mean.

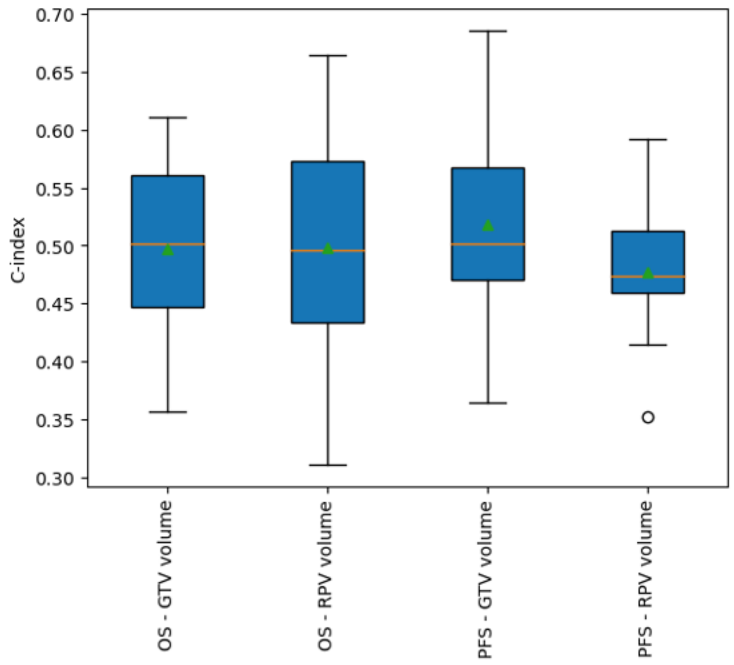

Figure S4: Box plot showing the results of the volume models (training-validation dataset 10-fold Cross Validation). The blue central boxes represent the interquartile range, with the median indicated by horizontal orange lines inside. Whiskers extend to the maximum non-outlier data points, circles denote outliers, and green triangles indicate the mean.

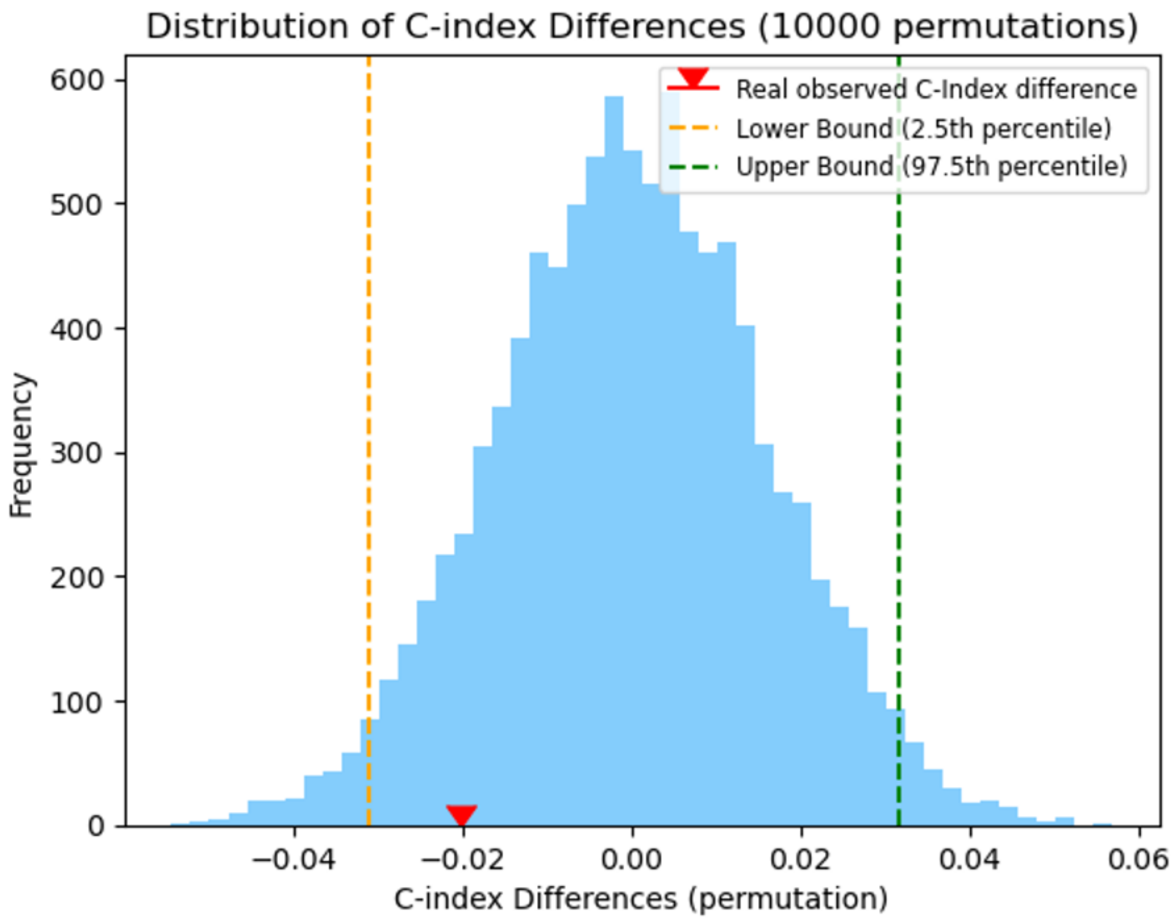

Figure S5: Histogram of two-sided permutation test for comparison between Clinical model and Clinical&GTV1 model for OS prediction. The result highlights that there is no statistically significant difference (Real observed difference between C-indexes: -0.02; p-value: 0.212).

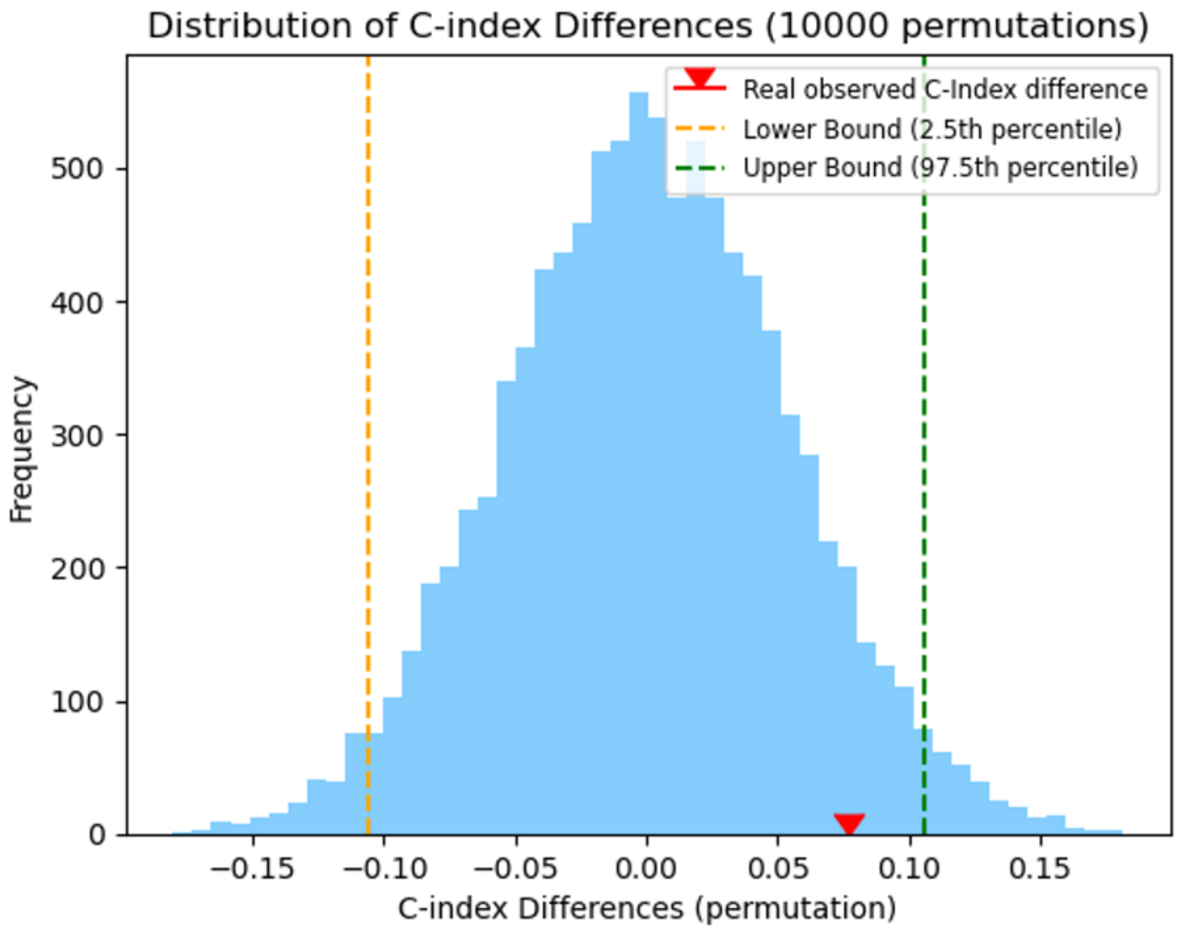

Figure S6: Histogram of two-sided permutation test for comparison between Clinical model and GTV1 radiomics model for OS prediction. The result highlights that there is no statistically significant difference (Real observed difference between C-indexes: -0.077; p-value: 0.156).

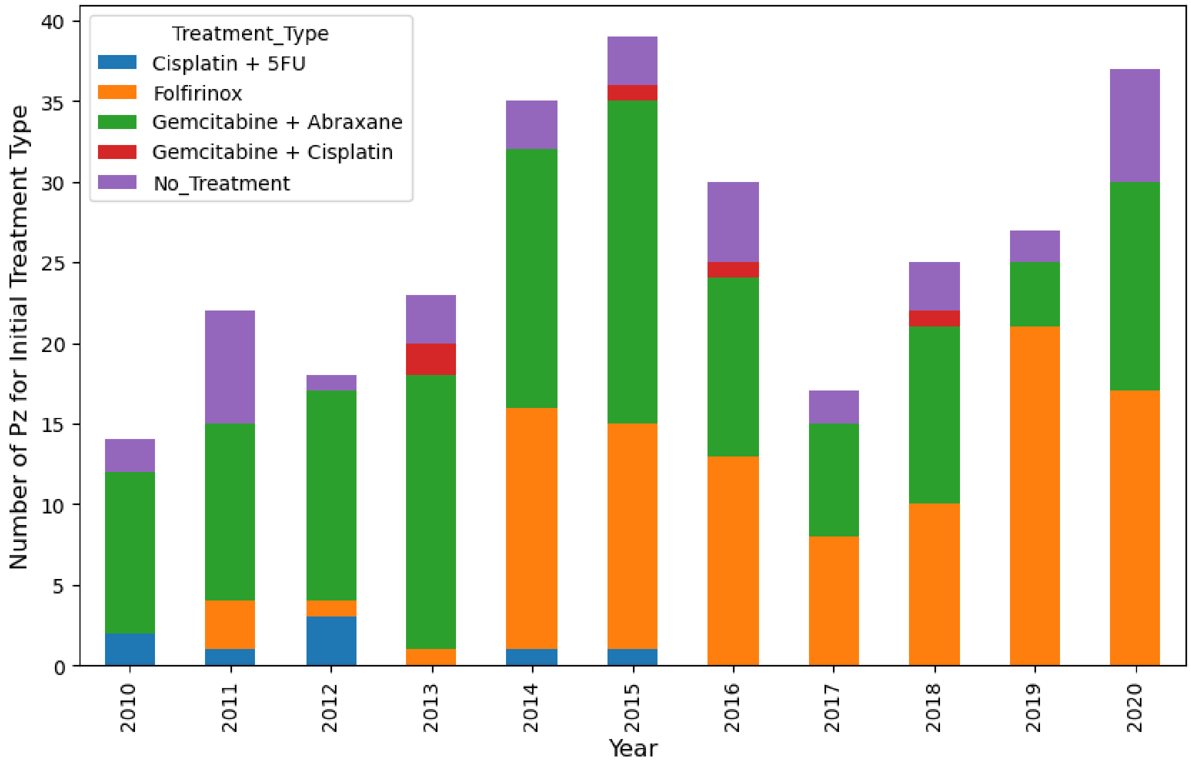

Figure S7: The treatment type, categorised by year and patient count.

Clinical, radiological and histological data

For each included patient the following clinical features data were collected:

- Demographic: age, gender; BMI, alcohol consumption (according to 5 classes: No consumption, <1 unit/day, 1 – 2/day, >3/day, or stopped consumption) and tobacco consumption (according to 4 classes: No consumption, <20 cigarettes/day op <15 Pack-year (PY), >20 cigarettes/day op >15PY, and old consumer).
- Clinical: tumour markers (Ca19.9 kU/L); treatment type (*); treatment strategy (*); Overall Survival (OS) in months (OS was computed from time of histological diagnosis to date of death; the date of death was achieved through the patient's electronic medical record; patients were considered censored if they were lost during follow-up); Progression Free Survival (PFS) in months (PFS was computed from time of treatment initiation to disease progression; patients were considered censored if they were lost during follow-up).
- Radiological: date of pretreatment contrast enhanced CT; location (pancreas head, body or tail); presence of metastatic disease. According to NCCN guidelines (version 2.2022[1,2]), patients were categorised into 4 subgroups (it was added a fourth group for metastatic patients):
  1 – Resectable;
  2 – Borderline resectable
  3 – Locally advanced unresectable
  4 – Metastatic
- Histological data: histological grade (well differentiated, moderately/poorly differentiated, or undifferentiated) and resection margins (only for surgically treated patients; if the patient had surgery without resection, it has been reported as “open-closed”).

(*) Treatment type and treatment strategy were not included as a clinical feature in the models. The treatment type, categorised by year and patient count, referring to the initial frontline chemotherapy treatment, is presented in Supplementary Materials Figure S7.

Ordinal encoding for clinical data

Gender: “Men” encoded as 1; “Women” encoded as 0.
Tobacco history: “No smoking” encoded as 0; “Stopped” encoded as 1; “<20 cigarette/day or <15 PY” encoded as 2; “>20 cigarette/day or >15 PY” encoded as 3.
Alcohol consumption: “No consumption” encoded as 0; “Old consumption” encoded as 1; “<1 unit/day” encoded as 2; “1-2 unit/day” encoded as 3; “>3 unit/day” encoded as 4.
Localisation of the lesions: “Head” encoded as 0; “Body” encoded as 1; “Tail” encoded as 2.

Subgroup: “Resectable” encoded as 0; “Borderline Resectable” encoded as 1; “Unresectable” encoded as 2; “Metastatic” encoded as 3.
R_margins: “Resection margin R0” encoded as 0, “Resection margin R1-R2” encoded as 1; “Open-closed without resection” encoded as 2; “No surgery” encoded as 3.
Histological grade: “Well differentiated” encoded as 0; “Moderately differentiated” encoded as 1; “Poorly differentiated” encoded as 2.

Patient Exclusion Criteria

The following table S1 presents additional details for the 20 patients who were not selected due to technical issues.

**Number of patients**	**Technical problem**
7	Data corruption
13	Irregular isotropic resolution

Table S1: Details regarding technical problems for 20 cases.

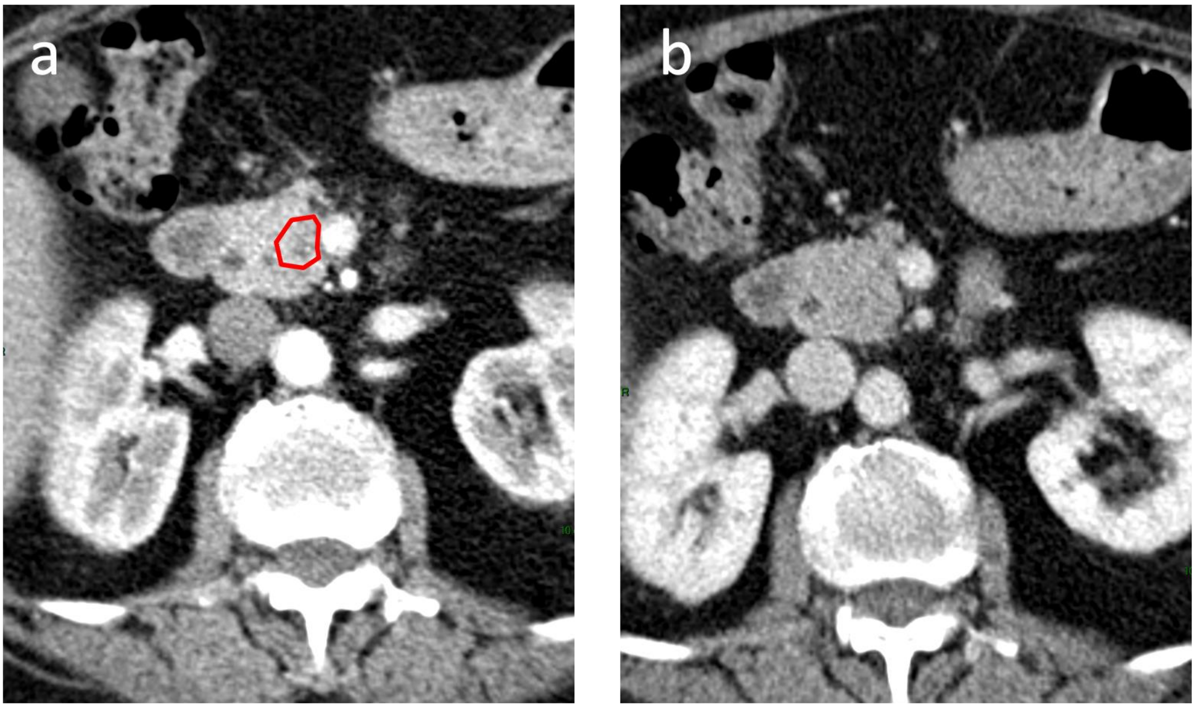

Figure S8: Image (**a**) displays a localised PDAC in the head of the pancreas (identified by segmentation with a red-coloured outline), visible in the arterial phase. Image (**b**) depicts the same slice in the portal phase; the lesion localised in the head of the pancreas is not clearly visible.

GradientBoostingSurvivalAnalysis classifier setting

GradientBoostingSurvivalAnalysis(n_estimators = 100, learning_rate = 0.001, max_depth = 4, min_samples_split = 10, min_samples_leaf = 2, max_features = 1, random_state = 0)

Assessment of feature repeatability

To evaluate the consistency of features for GTV1, as well as the reliability between the same rater (intra-rater) and different observers (inter-observer), GTV segmentations were independently performed a second time (GTV2) by the same radiologist, and a third time (GTV3) by a first-year radiologist resident on a subset of 45 randomly selected patients. The assessment of intra-rater and inter-observer reliability was conducted using the IntraClass Correlation Coefficient (ICC2) with a two-way mixed effects model for single measurements. Only features with an ICC2 value greater than 0.75 were considered for further analysis. Table S2 displays the ICC2 value for each individual radiomics feature.

Feature	ICC2
original_shape_Elongation	0.459575
original_shape_Flatness	0.458395
original_shape_LeastAxisLength	0.889306
original_shape_MajorAxisLength	0.795244
original_shape_Maximum2DDiameterColumn	0.784582
original_shape_Maximum2DDiameterRow	0.836128
original_shape_Maximum2DDiameterSlice	0.859434
original_shape_Maximum3DDiameter	0.803635
original_shape_MeshVolume	0.924731
original_shape_MinorAxisLength	0.846515
original_shape_Sphericity	0.118551
original_shape_SurfaceArea	0.890514
original_shape_SurfaceVolumeRatio	0.785983
original_shape_VoxelVolume	0.92473
original_firstorder_10Percentile	0.920356
original_firstorder_90Percentile	0.832288
original_firstorder_Energy	0.481856
original_firstorder_Entropy	0.862737
original_firstorder_InterquartileRange	0.846646
original_firstorder_Kurtosis	0.530602
original_firstorder_Maximum	0.50055
original_firstorder_MeanAbsoluteDeviation	0.765523
original_firstorder_Mean	0.880542
original_firstorder_Median	0.88733
original_firstorder_Minimum	0.424958
original_firstorder_Range	0.51967

original_firstorder_RobustMeanAbsoluteDeviation	0.849992
original_firstorder_RootMeanSquared	0.810483
original_firstorder_Skewness	0.472317
original_firstorder_TotalEnergy	0.481856
original_firstorder_Uniformity	0.855454
original_firstorder_Variance	0.202819
original_glcm_Autocorrelation	0.138281
original_glcm_ClusterProminence	0.016634
original_glcm_ClusterShade	0.03345
original_glcm_ClusterTendency	0.135008
original_glcm_Contrast	0.812275
original_glcm_Correlation	0.850655
original_glcm_DifferenceAverage	0.983567
original_glcm_DifferenceEntropy	0.982712
original_glcm_DifferenceVariance	0.489319
original_glcm_Id	0.994525
original_glcm_Idm	0.994789
original_glcm_Idmn	0.686105
original_glcm_Idn	0.799461
original_glcm_Imc1	0.914516
original_glcm_Imc2	0.859136
original_glcm_InverseVariance	0.983797
original_glcm_JointAverage	0.335886
original_glcm_JointEnergy	0.911571
original_glcm_JointEntropy	0.941204
original_glcm_MCC	0.818961
original_glcm_MaximumProbability	0.898468
original_glcm_SumAverage	0.335886
original_glcm_SumEntropy	0.812685
original_glcm_SumSquares	0.173292
original_gldm_DependenceEntropy	0.644118
original_gldm_DependenceNonUniformity	0.91154
original_gldm_DependenceNonUniformityNormalized	0.993398
original_gldm_DependenceVariance	0.982072
original_gldm_GrayLevelNonUniformity	0.952885
original_gldm_GrayLevelVariance	0.203357
original_gldm_HighGrayLevelEmphasis	0.143272
original_gldm_LargeDependenceEmphasis	0.994506
original_gldm_LargeDependenceHighGrayLevelEmphasis	0.294853
original_gldm_LargeDependenceLowGrayLevelEmphasis	0.724299
original_gldm_LowGrayLevelEmphasis	0.672107
original_gldm_SmallDependenceEmphasis	0.987764
original_gldm_SmallDependenceHighGrayLevelEmphasis	0.296193
original_gldm_SmallDependenceLowGrayLevelEmphasis	0.529537
original_glrlm_GrayLevelNonUniformity	0.940368
original_glrlm_GrayLevelNonUniformityNormalized	0.856519
original_glrlm_GrayLevelVariance	0.145011
original_glrlm_HighGrayLevelRunEmphasis	0.143642
original_glrlm_LongRunEmphasis	0.991854
original_glrlm_LongRunHighGrayLevelEmphasis	0.321922
original_glrlm_LongRunLowGrayLevelEmphasis	0.654944

original_glrlm_LowGrayLevelRunEmphasis	0.662079
original_glrlm_RunEntropy	0.803156
original_glrlm_RunLengthNonUniformity	0.876233
original_glrlm_RunLengthNonUniformityNormalized	0.994128
original_glrlm_RunPercentage	0.994718
original_glrlm_RunVariance	0.992352
original_glrlm_ShortRunEmphasis	0.991254
original_glrlm_ShortRunHighGrayLevelEmphasis	0.178362
original_glrlm_ShortRunLowGrayLevelEmphasis	0.631633
original_glszm_GrayLevelNonUniformity	0.889301
original_glszm_GrayLevelNonUniformityNormalized	0.661467
original_glszm_GrayLevelVariance	0.263403
original_glszm_HighGrayLevelZoneEmphasis	0.163298
original_glszm_LargeAreaEmphasis	0.961113
original_glszm_LargeAreaHighGrayLevelEmphasis	0.609591
original_glszm_LargeAreaLowGrayLevelEmphasis	0.857048
original_glszm_LowGrayLevelZoneEmphasis	0.561977
original_glszm_SizeZoneNonUniformity	0.879926
original_glszm_SizeZoneNonUniformityNormalized	0.969058
original_glszm_SmallAreaEmphasis	0.963481
original_glszm_SmallAreaHighGrayLevelEmphasis	0.216827
original_glszm_SmallAreaLowGrayLevelEmphasis	0.371869
original_glszm_ZoneEntropy	0.753702
original_glszm_ZonePercentage	0.974109
original_glszm_ZoneVariance	0.961093
original_ngtdm_Busyness	0.871962
original_ngtdm_Coarseness	0.876649
original_ngtdm_Complexity	0.271338
original_ngtdm_Contrast	0.825409
original_ngtdm_Strength	0.262095

Table S2: ICC2 values for radiomics features.

Independent features

To reduce redundancy and eliminate highly correlated features, we applied a Spearman correlation coefficient threshold of 0.80. The resulting table S3 presents the sets of not correlated features that were deemed reliable and informative both for OS and PFS:

Clinical features	GTV1	RPV	clinical&GTV1	clinical&GTV1&RPV
Localization<br>Gender<br>Age<br>BMI<br>Alcohol_quant<br>Tobacco_quant<br>CA19<br>Subgroup<br>Grading<br>R_Margins	original_shape_LeastAxisLength_GTV<br>original_firstorder_10Percentile_GTV<br>original_firstorder_90Percentile_GTV<br>original_firstorder_Entropy_GTV<br>original_glcm_Contrast_GTV<br>original_glcm_Correlation_GTV<br>original_glcm_Idn_GTV<br>original_glcm_InverseVariance_GTV	original_shape_Elongation_RPV<br>original_shape_Flatness_RPV<br>original_shape_LeastAxisLength_RPV<br>original_shape_MajorAxisLength_RPV<br>original_shape_Maximum2DDiameterColumn_RPV<br>original_shape_Maximum2DDiameterRow_RPV<br>original_shape_Maximum2DDiameterSlice_RPV<br>original_shape_MeshVolume_RPV<br>original_shape_MinorAxisLength_RPV<br>original_shape_Sphericity_RPV<br>original_shape_SurfaceArea_RPV<br>original_shape_SurfaceVolumeRatio_RPV<br>original_firstorder_10Percentile_RPV<br>original_firstorder_90Percentile_RPV<br>original_firstorder_Energy_RPV<br>original_firstorder_Entropy_RPV<br>original_firstorder_Kurtosis_RPV<br>original_firstorder_Maximum_RPV<br>original_firstorder_Minimum_RPV<br>original_firstorder_Range_RPV<br>original_firstorder_Skewness_RPV<br>original_glcm_ClusterShade_RPV<br>original_glcm_Contrast_RPV<br>original_glcm_Correlation_RPV<br>original_glcm_Idmn_RPV<br>original_glcm_InverseVariance_RPV<br>original_gldm_LargeDependenceHighGrayLevelEmphasis_RPV<br>original_glszm_LargeAreaEmphasis_RPV<br>original_ngtdm_Busyness_RPV	Localization<br>Gender<br>Age<br>BMI<br>Alcohol_quant<br>Tobacco_quant<br>CA19<br>Subgroup<br>Grading<br>R_Margins<br>original_shape_LeastAxisLength_GTV<br>original_firstorder_10Percentile_GTV<br>original_firstorder_90Percentile_GTV<br>original_firstorder_Entropy_GTV<br>original_glcm_Contrast_GTV<br>original_glcm_Correlation_GTV<br>original_glcm_Idn_GTV<br>original_glcm_InverseVariance_GTV	Localization<br>Gender<br>Age<br>BMI<br>Alcohol_quant<br>Tobacco_quant<br>CA19<br>Subgroup<br>Grading<br>R_Margins<br>original_shape_LeastAxisLength_GTV<br>original_firstorder_10Percentile_GTV<br>original_firstorder_90Percentile_GTV<br>original_firstorder_Entropy_GTV<br>original_glcm_Contrast_GTV<br>original_glcm_Correlation_GTV<br>original_glcm_Idn_GTV<br>original_glcm_InverseVariance_GTV<br>original_shape_Elongation_RPV<br>original_shape_Flatness_RPV<br>original_shape_LeastAxisLength_RPV<br>original_shape_MajorAxisLength_RPV<br>original_shape_Maximum2DDiameterColumn_RPV<br>original_shape_Maximum2DDiameterRow_RPV<br>original_shape_Maximum2DDiameterSlice_RPV<br>original_shape_MeshVolume_RPV<br>original_shape_MinorAxisLength_RPV<br>original_shape_Sphericity_RPV<br>original_shape_SurfaceArea_RPV<br>original_shape_SurfaceVolumeRatio_RPV<br>original_firstorder_10Percentile_RPV<br>original_firstorder_90Percentile_RPV<br>original_firstorder_Energy_RPV<br>original_firstorder_Entropy_RPV<br>original_firstorder_Kurtosis_RPV<br>original_firstorder_Maximum_RPV<br>original_firstorder_Minimum_RPV<br>original_firstorder_Range_RPV<br>original_firstorder_Skewness_RPV<br>original_glcm_ClusterShade_RPV<br>original_glcm_Correlation_RPV<br>original_glcm_Idmn_RPV<br>original_glcm_InverseVariance_RPV<br>original_gldm_LargeDependenceHighGrayLevelEmphasis_RPV<br>original_glszm_LargeAreaEmphasis_RPV<br>original_ngtdm_Busyness_RPV

Table S3: List of not correlated features.

Univariate analysis

The table S4 presents the results of the univariate analysis for each individual clinical and radiomic feature used in the models. It specifically reports the corresponding C-indexes obtained from the GradientBoostingSurvivalAnalysis classifier trained on the training-validation dataset and tested on the testing dataset.

Feature (OS prediction)	C-index	Feature (PFS prediction)	C-index
Subgroup_Clinical	0.662307	Subgroup_Clinical	0.668176
CA19_Clinical	0.597479	CA19_Clinical	0.536108
R_Margins_Clinical	0.628413	R_Margins_Clinical	0.630722
Grading_Clinical	0.496086	Localization_Clinical	0.612607
Age_Clinical	0.503342	Age_Clinical	0.528519
original_firstorder_90Percentile_GTV	0.626122	Alcohol_quant_Clinical	0.579315
original_glcm_ClusterShade_RPV	0.479282	Gender_Clinical	0.497062
original_firstorder_Skewness_RPV	0.555566	Tobacco_quant_Clinical	0.482864
original_shape_Sphericity_RPV	0.516231	Grading_Clinical	0.639657
original_glcm_InverseVariance_RPV	0.499427	original_glcm_Contrast_GTV	0.508201
original_shape_Flatness_RPV	0.514989	original_glcm_Contrast_RPV	0.498898
original_glcm_Contrast_RPV	0.491598	original_glcm_Correlation_RPV	0.533782
original_shape_LeastAxisLength_RPV	0.509547	original_firstorder_90Percentile_GTV	0.562668
original_shape_MeshVolume_GTV	0.582490	original_glcm_Correlation_GTV	0.573072
original_shape_MeshVolume_RPV	0.438037	original_glcm_Idn_GTV	0.564015
		original_shape_MeshVolume_GTV	0.511506
		original_shape_MeshVolume_RPV	0.538066

Table S4: Univariate analysis for clinical and radiomic features.

C-Indexes across different types of treatment

The following table S5 presents the results for the c-index in predicting OS and PFS using top-performing models for various patient subgroups with different types of treatment. In particular, a fairness analysis was applied. To obtain reliable results, the analysis was conducted only on subgroups with more than 30 patients and a censored patient count below 80%[3]. The analysis utilised 10-fold cross-validation on the entire dataset.

	Type of treatment	Number of patients	Censored patients (Percentage)	C-index
OS prediction	FOLFIRINOX	103	15%	0.730
	Gemcitabine + Abraxane	133	12%	0.701
	Cisplatin + 5-Fluorouracil	8	12%	-
	Gemcitabine + Cisplatin	5	20%	-
	No treatment	38	11%	0.701
	All	287	13%	0.711
PFS prediction	FOLFIRINOX	103	17%	0.694
	Gemcitabine + Abraxane	133	20%	0.680
	Cisplatin + 5-Fluorouracil	8	38%	-
	Gemcitabine + Cisplatin	5	0%	-
	No treatment	38	82%	-
	All	287	27%	0.677

Table S5: C-indexes in predicting OS and PFS for various patient subgroups with different types of treatment.

Intercorrelation between chosen features and volumes.

	GTV1 volume	RPV volume
Subgroup_Clinical	0.39	0.13
CA19_Clinical	0.24	-0.11
R_Margins_Clinical	0.40	0.04
Grading_Clinical	0.01	0.07
Age_Clinical	-0.10	-0.20
original_firstorder_90Percentile_GTV	-0.22	-0.09
original_glcm_ClusterShade_RPV	0.08	0.08
original_firstorder_Skewness_RPV	0.16	-0.20
original_shape_Sphericity_RPV	-0.69	0.03
original_glcm_InverseVariance_RPV	-0.04	-0.13
original_shape_Flatness_RPV	0.49	0.03
original_glcm_Contrast_RPV	-0.13	-0.20
original_shape_LeastAxisLength_RPV	0.55	0.67
Localization_Clinical	0.41	0.02
Alcohol_quant_Clinical	-0.09	-0.00
Gender_Clinical	0.08	0.20
Tobacco_quant_Clinical	-0.05	0.19
original_glcm_Contrast_GTV	-0.08	-0.04

original_glcm_Correlation_RPV	0.13	-0.10
original_glcm_Correlation_GTV	0.31	-0.16
original_glcm_Idn_GTV	0.60	-0.09
GTV1 volume	1.00	0.18
RPV volume	0.18	1.00

Table S6: The intercorrelation between the chosen features and volumes in the testing dataset (missing clinical features were filled using the MissForest package; the values were not standardised). The volumes for GTV1 and RPV are calculated using the triangle mesh method [4,5]. The Spearman correlation coefficient was utilised to calculate the inter-correlation among the selected features and volumes.

Radiomics quality score

The radiomics workflow was assessed using the Radiomics Quality Score (RQS) [6,7]. The RQS score obtained for this study was 17/36. The RQS is determined by a 36-point system. A higher score indicates a greater level of methodological quality in reporting and research[7].

References

1. Zins, M.; Matos, C.; Cassinotto, C. Pancreatic Adenocarcinoma Staging in the Era of Preoperative Chemotherapy and Radiation Therapy. *Radiology* **2018**, *287*, 374–390.
2. Tempero, M.A.; Malafa, M.P.; Al-Hawary, M.; Behrman, S.W.; Benson, A.B.; Cardin, D.B.; Chiorean, E.G.; Chung, V.; Czito, B.; Del Chiaro, M.; et al. Pancreatic Adenocarcinoma, Version 2.2021, NCCN Clinical Practice Guidelines in Oncology. *J. Natl. Compr. Canc. Netw.* **2021**, *19*, 439–457.
3. Zhu, X.; Zhou, X.; Zhang, Y.; Sun, X.; Liu, H.; Zhang, Y. Reporting and Methodological Quality of Survival Analysis in Articles Published in Chinese Oncology Journals. *Medicine* **2017**, *96*, e9204.
4. Lorensen, W.E.; Cline, H.E. Marching Cubes: A High Resolution 3D Surface Construction Algorithm. *Comput. Graph.* **1987**, *21*, 163–169.
5. van Griethuysen, J.J.M.; Fedorov, A.; Parmar, C.; Hosny, A.; Aucoin, N.; Narayan, V.; Beets-Tan, R.G.H.; Fillion-Robin, J.-C.; Pieper, S.; Aerts, H.J.W.L. Computational Radiomics System to Decode the Radiographic Phenotype. *Cancer Res.* **2017**, *77*, e104–e107.
6. Lambin, P.; Leijenaar, R.T.H.; Deist, T.M.; Peerlings, J.; de Jong, E.E.C.; van Timmeren, J.; Sanduleanu, S.; Larue, R.T.H.M.; Even, A.J.G.; Jochems, A.; et al. Radiomics: The Bridge between Medical Imaging and Personalized Medicine. *Nat. Rev. Clin. Oncol.* **2017**, *14*, 749–762.
7. Sanduleanu, S.; Woodruff, H.C.; de Jong, E.E.C.; van Timmeren, J.E.; Jochems, A.; Dubois, L.; Lambin, P. Tracking Tumor Biology with Radiomics: A Systematic Review Utilizing a Radiomics Quality Score. *Radiother. Oncol.* **2018**, *127*, 349–360.
